# Short cervix and use of cervical pessary for preventing preterm birth in singleton and twin pregnancies: a systematic review and meta-analysis

**DOI:** 10.61622/rbgo/2025rbgo10

**Published:** 2025-03-17

**Authors:** Ana Clara Felix de Farias Santos, Nicole dos Santos Pimenta, Ana Gabriela Alves Pereira, Gabriela Oliveira Gonçalves Molino, Maírla Marina Ferreira Dias, Pedro Henrique Costa Matos da Silva

**Affiliations:** 1 Universidade Cidade de São Paulo São Paulo SP Brazil Universidade Cidade de São Paulo, São Paulo, SP, Brazil.; 2 Universidade Federal do Estado do Rio de Janeiro Rio de Janeiro RJ Brazil Universidade Federal do Estado do Rio de Janeiro, Rio de Janeiro, RJ, Brazil.; 3 Universidade Estadual Paulista São Paulo SP Brazil Universidade Estadual Paulista, São Paulo, SP, Brazil.; 4 Universidade Federal de Ciências da Saúde de Porto Alegre Porto Alegre RS Brazil Universidade Federal de Ciências da Saúde de Porto Alegre, Porto Alegre, RS, Brazil.; 5 Universidade Federal de Campina Grande Campina Grande PB Brazil Universidade Federal de Campina Grande, Campina Grande, PB, Brazil.; 6 Universidade Federal de Goiás Department of Gynecologic and Obstetrics Goiânia GO Brazil Department of Gynecologic and Obstetrics, Universidade Federal de Goiás, Goiânia, GO, Brazil.

**Keywords:** Cervical length measurement, Pessaries, Progesterone, Premature birth, Short cervix

## Abstract

**Objective:**

Preterm birth remains a significant contributor to neonatal morbidity and mortality. The use of cervical pessaries as an intervention for preventing preterm delivery in women with a short cervix has been a subject of interest. We evaluated the effectiveness of cervical pessary compared to standard care in preventing preterm delivery in women with a short cervix.

**Data source:**

Databases were systematically searched in PubMed, Cochrane, and Embase databases in December 2023.

**Study selection:**

Randomized clinical trials with the outcomes of interest were included.

**Data collect:**

We computed risk ratios for binary endpoints, with 95% confidence intervals. Heterogeneity was assessed using I2 statistics. Data were analyzed using R software (version 4.3.0). The primary outcomes of interest were preterm delivery before 37 weeks, and preterm delivery before 34 weeks.

**Data synthesis:**

Seventeen studies with 5,704 patients were included. The use of cervical pessary was associated with a decreased risk of preterm delivery before 37 (RR 0.88; 95% CI 0.81-0.96) and 34 weeks (RR 0.79; 95% CI 0.63-0.99) of gestation in twin pregnancies as compared to standard care without progesterone. There were no significant differences in preterm delivery in singleton pregnancy, neonatal outcomes, preterm premature rupture of the membranes or chorioamnionitis.

**Conclusion:**

The use of cervical pessary was associated with a significant reduction in preterm delivery at 34 and 37 weeks of gestation in twin pregnancies among patients with a short cervix compared to no treatment. No significant difference was found in singleton pregnancies or maternal outcomes.

## Introduction

Preterm birth, defined as birth occurring before 37 completed weeks of gestation, has a significant impact on both global and domestic levels. It is estimated that approximately 15 million infants are born preterm worldwide, with a particular burden on low- and middle-income countries.^(1–25)^ In developed countries such as the United States, preterm birth affects around 1 in every 10 infants, while in Europe, reported rates generally range from 5% to 9%.^(26,27)^ Preterm births are responsible for 75% of perinatal mortality and contribute to over half of the long-term morbidity cases. Surviving infants face an increased risk of neurodevelopmental impairments and experience respiratory and gastrointestinal complications.^(28–30)^ Although the exact mechanisms leading to preterm birth remain unclear, several factors have been identified as potential explanations. Infection or inflammation, uteroplacental ischemia or hemorrhage, uterine overdistension, stress, and other immunologically mediated processes are among the factors that could potentially trigger preterm labor.^(29,30)^

Shortened cervical length is a crucial factor of spontaneous preterm birth; clinicians often rely on the safe and effective method of using a cervical pessary to prevent preterm birth, particularly in women with a shortened cervix.^(31,32)^ However, patients with a short cervix and different types of pregnancies, such as twins, experienced mixed effects in the use of pessary for preventing spontaneous preterm birth.^(5,13)^ Due to the significant inconsistency in these results, multiple randomized controlled trials (RCTs) have been conducted to compare cervical pessary insertion and standard care in pregnant women with singleton or twin pregnancy, with the cervical pessary group consistently reporting no inferior outcomes in singleton pregnancies.^(2,4,8)^

Previous meta-analyses with RCTS and retrospective studies have been performed. The most recent one incorporated data from observational and randomized studies conducted until 2021.^(33)^ However, since then, more comprehensive RCTs have been published, involving a larger number of patients and employing stronger methodological approaches compared to the meta-analysis.^(8)^ Therefore, this systematic review and meta-analysis aimed to compare the effectiveness of cervical pessary versus standard care in patients with a short cervix, assessing whether there is a practical advantage or not from the cervical pessary treatment.

## Methods

### Eligibility criteria

Two authors independently conducted literature searches and assessed the titles, abstracts, and full papers of the selected references. Studies that met the following eligibility criteria were included: 1) RCT; 2) comparison of cervical pessary with standard care; 3) enrolment of patients with short cervix defined as 25 mm or less for singleton pregnancies, and for twin pregnancies without a specific cut-off point, using data based on percentiles for inclusion; 4) reporting at least one outcome of interest. We excluded overlapping populations, defined as studies with overlapping institutions and recruitment periods, non-randomized studies, and studies that unified singleton and twin pregnancies.

### Search strategy and data extraction

We systematically searched PubMed, Embase and Cochrane Central register of Controlled trials databases for studies meeting the eligibility criteria and published up to December 2023. The search strategy included the terms "short cervix" and "cervical pessary", along with their synonyms. A comprehensive search strategy is available in the Supplementary table 1S. Additionally, we analyzed the references of systematic reviews and included studies to identify any other potentially eligible studies. Two investigators independently extracted prespecified baseline characteristics and outcome data. Disagreements were resolved by consensus between two authors and the senior author after checking the reasons for any discrepancies. Data on the standard care definition available in the RCTs were extracted for analysis. Outcomes data were cross-checked, combined, and inputted in the meta-analysis software. The protocol for this research was submitted to the International Prospective Register of Systematic Reviews (PROSPERO) with registration number CRD42024499300. The systematic review and meta-analysis followed the recommendations of the Preferred Reporting Items for Systematic Reviews and Meta-Analysis (PRISMA) statement guidelines.^(18)^

### Endpoints and subgroup analysis

Primary outcomes of interest were: The primary outcomes of interest included: 1) preterm delivery before 37 weeks, and 2) preterm delivery before 34 weeks. Secondary outcomes of interest were: 3) chorioamnionitis; 4) preterm prelabor rupture of membranes (PPROM); 5) vaginal discharge; and 6) neonatal outcomes. We performed two subgroup analyses for the primary outcomes of preterm delivery before 37 and 34 weeks. First, we investigated the effectiveness of cervical pessary compared to vaginal progesterone, as this was the most common standard care in studies with singleton pregnancy. Second, in the primary endpoints we also assessed the effects of influential studies on the pooled results by sequentially removing one study's data and re-analyzing the remaining data (leave-one-out analysis) to preserve the stability of the pooled treatment effect. Study dominance was assigned whenever the omission of a study shifted the pooled effect size p-values from significant to non-significant, or vice-versa.^(23)^

### Quality assessment and publication bias

The Cochrane Collaboration tool for assessing risk of bias in randomized trials (RoB 2) was used to assess the quality of individual RCTs.^(19)^ Two independent investigators conducted the quality assessment. Each trial received a score of high, low or unclear risk of bias in five domains: randomization process; deviations from the intended interventions; missing outcomes; measurement of the outcome; and selection of reported results. The layout was created by Robvis.^(20)^ Potential publication bias was evaluated by visually examining funnel plots and analyzing the control lines.

### Statistical analysis

The treatment effects for binary endpoints were compared using risk ratio (RR), with 95% confidence intervals (CIs). Heterogeneity was assessed with the Cochrane Q-test and I^2^ statistics; P values > 0.10 and I^2^ values > 25% were considered to indicate significance for heterogeneity.^(21)^ Mantel-Haenszel and restricted maximum likelihood estimator were used in all outcomes with low or significant heterogeneity. Random effects meta-analyses were used to calculate pooled effect sizes incorporating the following assumptions: the studies included various control types, and the trials were conducted with diverse patient inclusion or exclusion criteria, which could indicate variations in care. Hence, the variation among the different effect estimates may be accounted for by within-study sampling error, between-study heterogeneity, or a combination of both factors.^(24)^ For data handling, we used the guidelines of the Cochrane Handbook for Systematic Reviews of Interventions.^(22)^ Statistical analyses were performed using the R software environment, version 4.3.0 (R Foundation for Statistical Computing).

## Results

### Study selection and characteristics

**Figure 1:**
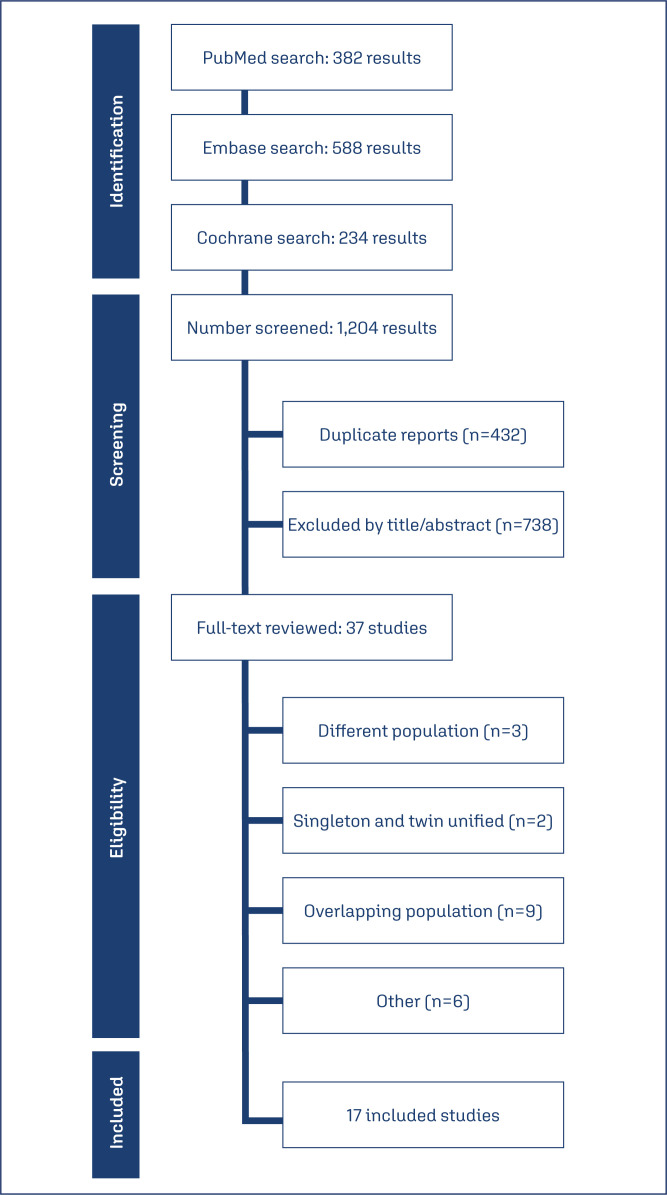
Study screening and selection

As illustrated in figure 1, 1,204 studies were identified. After removal of duplicates, non-RCTs and non-relevant studies, 37 articles remained. These articles were rigorously reviewed to ensure they met the inclusion criteria. Articles were excluded if they conducted combined analyses of singleton and twin pregnancies without separate assessments or if they had overlapping populations. Seventeen manuscripts met all inclusion criteria and were included in quantitative analyses. The baseline characteristics in individual studies were mostly comparable between groups. A non-overlapping population of 5,704 patients was included, of whom 2,891(50.6%) received cervical pessary treatment. The mean age ranged from 27 to 37 years, and the mean BMI ranged from 20.9 to 28.8 kg/m^2^. The median or mean cervical length ranged from 10 to 35 mm. All studies in singleton pregnancy included women with a cervical length of 25 mm or less. However, in twin pregnancy studies, there was some discrepancy in the definition of a short cervix. Only two studies used the threshold of 25mm or less for a short cervix.^(5,12)^ The other six studies defined a short cervix as 38mm or less.^(1,3,7,10,13,15)^ Additionally, four studies identified subgroups with even shorter cervix lengths.^(5,12,13,15)^ Study characteristics are presented in chart 1.

**Chart 1:** Baseline characteristics of included studies

Study	Country	Type of pregnancy	Intervention	Control	N° of patients CP/C	GA † * (wk) CP/C	CL† * (mm) CP/C	Age† § (y) CP/C	BMI† (kg/m^2^) CP/C	Nulliparous (n) CP/C
Berghella et al. (2017^)(1^)	USA	Twin	CP	NA	23/23	21.0/ 21.2	16.7/ 22.9	27.0/32.9	24.7/28.2^c^	11/ 15
Cruz-Melguizo et al. (2018^)(2^)	Spain	Singleton	CP	VP	127/119	21.2/ 21.3	20.8/ 20.9	32.5/33.1	24.4/ 23.7	58/54
Dang et al. (2019^)(3^)	Vietnam	Twin	CP	VP	150/150	17.5/ 18.0	30.9/31.7	31.7/32.1	21.2/20.9	125/ 135
Dugoff et al. (2018^)(4^)	USA	Singleton	CP + VP	VP	60/58	20.9/ 20.7	17.6/ 19.0	27.7/ 29.5	25.6/ 24.6^c^	38/ 40
Goya et al. (2016^)(5^)	Spain	Twin	CP	NA	68/66	22.1/22.5	19.2/19.6	35.4/35.9	24.3/24.7	31/29
Goya et al. (2012^)(6^)	Spain	Singleton	CP	NA	190/190	22.2/22.4	19.0/19.0	30.3/29.6	24.9/24.5	94/96
Groussolles et al. (2022^)(7^)	France	Twin	CP	NA	157/158	20.8/20.9	27.0/25.0	30.9/31.5	23.2/23.1	96/93
Hoffman et al. (2023^)(8^)	USA	Singleton	CP + VP	VP	280/264	21.7/21.9^g^	13.3/13.7^h^	29.9/29.1	28.8/28.6^f^	NA
Karbasian et al. (2016^)(9^)	Iran	Singleton	CP + VP	VP	71/73	19.7/19.5^g^	22.0/22.0	28.8/27.9	24.1/24.4	NA
Liem et al. (2013^)(10^)	Netherlands	Twin	CP	NA	401/407	16.9/17.0	35/34^j^	33.1/32.7^i^	23.7/22.9	222/225
Mastantuoni et al. (2021^)(11^)	Italy	Singleton	CP + VP	VP	32/29	28.9/ 30.6	<25/<25	30.5/ 31.1	25.8/ 26.1	20/23
Merced et al. (2019^)(12^)	Spain	Twin	CP	NA	67/65	28.0/29.0	10.0/11.0	37.0/36.0	24.3/24.1	30/28
Nicolaides et al. (2016)^(13)^	Europe + SA^a^	Twin	CP	NA	590/590	22.6/22.7	32.0/32.0	33.1/33.2	NA	363/360
Nicolaides et al. (2016)^(14)^	Europe^b^ + Chile	Singleton	CP + VP	VP	465/467	23.4/23.6	20.0/20.0	30.1/29.5	NA	248/257
Norman et al. (2021^)(15^)	Belgium + UK	Twin	CP	NA	250/253	NA	28.8/29.5	32.4/32.7	NA	107/99^m^
Pratcorona et al. (2018^)(16^)	Spain	Singleton	CP	NA	179/178	28.6/28.4	15.3/15.2	28.7/29.4	24.7/25.0	90/84
Saccone et al. (2017^)(17^)	Italy	Singleton	CP + VP	VP	150/150	22.3/22.4	11.5/12.5	28.5/28.9	26.7/26.4	104/105

BMI: body mass index; C, control; CL: cervical length; GA, gestational age in weeks; CP, Cervical Pessary; NA: not available; VP: vaginal progesterone.

† Mean or median;

§ Maternal age;

* At randomization

a The United Kingdom, Spain, Germany, Austria, Slovenia, Portugal, Italy, Belgium, Albania, China, Brazil, and Chile;

b England, Slovenia, Portugal, Australia, Italy, Albania, Germany, and Belgium

c Prepregnancy Body mass index,

d Smoker during pregnancy,

e Cigarette smoking during pregnancy >5 per day,

f Body mass index at first clinic visit,

g Gestation at enrollment,

h Cervical length at screening,

i Age at randomization for sample size of 813 patients including subgroups,

j Cervical length overall for subgroup,

k Cigarette smoking during pregnancy,

l Current smoker,

m No previous pregnancies

### Sub analysis in selected populations

#### Singleton pregnancy

**Figure 2:**
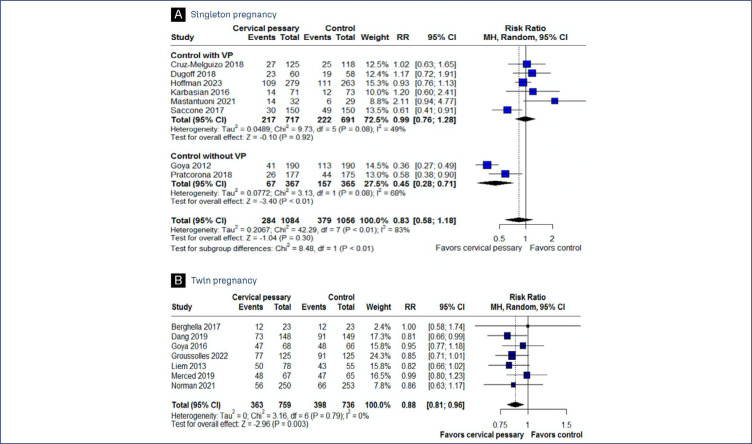
Forest plot of studies examining Preterm delivery < 37 weeks between patients undergoing cervical pessary or standard treatment. A: Singleton pregnancy; B: Twin pregnancy

**Figure 3:**
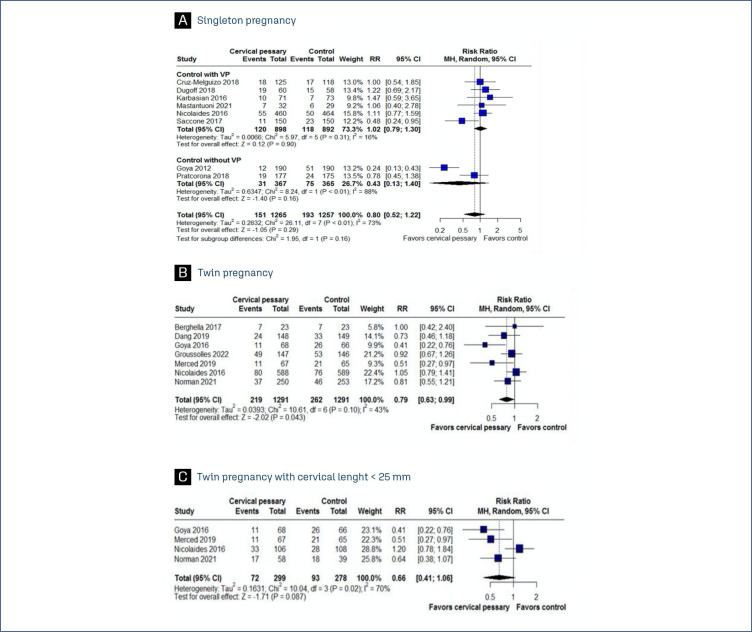
Forest plot of studies examining Preterm delivery < 34 weeks between patients undergoing cervical pessary or standard treatment. A: Singleton pregnancy; B: Twin pregnancy; C: Twin pregnancy with cervical length < 25mm

Preterm delivery before 37 weeks was significantly less frequent among patients receiving cervical pessary when compared to control group without progesterone. However, when directly comparing the cervical pessary intervention with progesterone treatment, we observed no statistically significant differences between the groups. No significant difference was observed in preterm delivery before 34 weeks between the groups (Figure 2A, 3A).

#### Twin pregnancy

In twin pregnancies, our analysis demonstrated a significantly lower incidence of preterm delivery before 37 weeks of gestation in patients treated with a cervical pessary, compared to the control groups. This trend was similarly observed for deliveries before 34 weeks of gestation. However, within the subgroup of patients with a cervical length of less than 25mm, no significant difference in the rate of preterm delivery before 34 weeks was identified between those treated with a cervical pessary and the control groups. It is important to note that, in the studies reviewed, the standard care provided to the control groups did not involve the administration of progesterone, which may influence the comparative interpretation of the results (Figure 2B, 3B, 3C).

#### Pooled analysis of all studies

Although the rate of vaginal discharge was higher among patients receiving cervical pessary, neonatal outcomes, PPROM and chorioamnionitis did not differ between groups in singleton and twin pregnancies subgroups (Supplementary Figure 1 and 2).

#### Sensitivity analysis

We performed a leave-one-out sensitivity analysis for preterm birth before 37 weeks in singleton and twin pregnancies subgroups. Overall, no change was observed in the statistical significance of outcome in each of the leave-one –out tests, for preterm birth before 37 weeks in twin pregnancy. This analysis is shown in supplementary figure 3. There was a significant reduction in heterogeneity among studies for the outcome of preterm birth before 37 weeks in singleton pregnancy with the removal of Goya et al.,^(6)^ with a reduction from I^2^=83% to I^2^=56%. This was likely attributable to the patient's characteristics in this study, which was substantially different than in the other trials.^(6)^ Otherwise, no significant major changes in the heterogeneity of the outcomes were observed when omitting each individual study in the leave-one-out analyses.

#### Risk of bias within studies and publication bias

**Figure 4:**
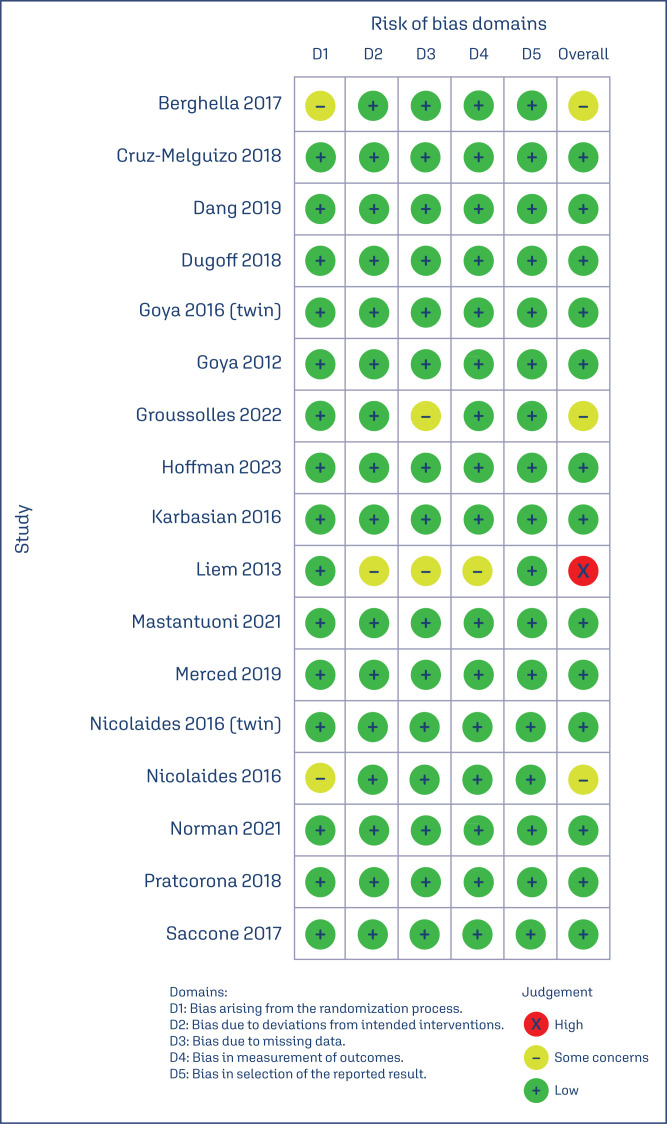
Critical appraisal of randomized controlled trials according to the Cochrane Collaboration tool for assessing risk of bias in randomized trials

The risk of individual within-study bias is represented in the RoB 2 traffic-light diagram (Figure 4). All studies were susceptible to performance bias, due to the impossibility of implementing patient and investigator blinding in the trials. Four RCTs raised some concerns about bias in at least one RoB 2 assessment tool domain.^(1,7,10,14)^ Of the thirteen remaining studies, all were assigned a low risk of bias.^(2–6,8,9,11–13,15,16)^

The analysis of the funnel plots of preterm birth before 37 weeks in singleton pregnancy suggests evidence of some publication bias, as some studies fall outside the control lines. For preterm birth before 37 weeks in twin pregnancy, the analysis of the funnel plots did not reveal significant evidence of serious publication bias. The funnel plots are available in supplemental figure 4.

## Discussion

In this systematic review and meta-analysis including seventeen studies and 5,704 patients, cervical pessary was compared with standard care with or without progesterone in patients with short cervix. The main findings were as follows: cervical pessary was associated with 1) a significant reduction in preterm delivery before 37 weeks in singleton pregnancy, control group without progesterone use; 2) a significant reduction in preterm delivery before 37 and before 34 weeks in twin pregnancy; and 3) no significant difference in preterm delivery before 34 weeks in twin pregnancy with short cervix <25mm, neonatal outcomes, PPROM and chorioamnionitis.

Currently, progesterone therapy is indicated as prevention of preterm birth in patients with short cervix or other risk factors for preterm labor.^(34–41)^ Some studies attempted to assess the effectiveness of cervical pessary in this patient population. A meta-analysis by Zhuang et al.^(33)^ found that patients receiving cervical pessary and vaginal progesterone included a greater proportion achieving preterm delivery before 34 weeks compared with those receiving vaginal progesterone alone. Furthermore, another meta-analysis comparing cervical pessary with no treatment or vaginal progesterone also showed a positive effect of cervical pessary on the risk reduction of delivery before 34 weeks or 37 weeks of gestation.^(42)^

Cervical pessary has also been shown to prolong gestation in twin pregnancies.^(43)^ The significant prolongation seen in outcome with cervical pessary may relate to: support provided to the cervix, enhancement of cervical length, and reduction of cervical funneling.^(44)^ Our meta-analysis further confirms these findings, particularly in preventing preterm delivery before 34 weeks and 37 weeks’ gestation. Additionally, a recent study hypothesized that cervical elastography with E-Cervix™ could be useful for assessing twin gestations presenting to obstetrics triage for threatened preterm labor.^(45,46)^

Short cervix is an established risk factor for preterm delivery. Cervical pessary may be effective in reducing preterm birth outcomes in this patient population.^(35)^ Two randomized clinical trials showed that women with singleton pregnancies and short transvaginal cervical length, use of a cervical pessary, compared with no pessary use resulted in a lower rate of spontaneous preterm birth at less than 34 weeks of gestation and prevented preterm birth.^(6,17)^

It is crucial to highlight that in singleton pregnancies, cervical pessary was significant only when compared to no treatment or the absence of progesterone use. However, when compared to vaginal progesterone, a well-established and recommended treatment for preventing preterm births in patients with a short cervix, the benefits of the cervical pessary were no longer significant.^(45)^ This observation is further supported by our subgroup analysis, which indicates that the advantageous effects of the pessary are predominantly significant when progesterone is not used, raising questions about its comparative efficacy.

Contrasting findings were shown with cervical pessary when analyzed in women with twin pregnancy. A study conducted by Norman et al.^(15)^ demonstrated insertion of cervical pessary did not help prevent preterm birth before 34 weeks of gestation, results being aligned with other 2 studies.^(1,13,15)^ However, a clinical trial conducted by Goya et al.^(5)^ proposed the use of a cervical pessary for preventing preterm birth in twin pregnancies of mothers with a short cervix due to insertion of a cervical pessary was associated with a significant reduction in spontaneous preterm birth rate, results consistent with a trial conducted by Liem et al.^(10)^ indicated that in a subgroup analysis of women with a cervical length of less than the 25^th^ percentile (<38mm), the pessary was shown to significantly reduce frequency of poor perinatal outcome and very preterm delivery.^(5,10)^

Importantly, our analysis revealed no significant difference between cervical pessary and standard care or progesterone in terms of PPROM. This finding is consistent with a prior meta-analysis that included a smaller patient population and similarly found no significant difference in PPROM when comparing cervical pessary with standard care.^(42)^

Currently, a few maternal side effects or adverse events of cervical pessary are documented, such as chorioamnionitis and vaginal discharge.^(35,47)^ This meta-analysis evaluated some of these adverse effects and the results showed no significant difference in chorioamnionitis outcomes between groups. The relative increased vaginal discharge is a commonly reported side effect of using a pessary, as fluid can accumulate behind it and be released through perforations.^(36,37)^ The risk of increased vaginal discharge was significant in previous studies with cervical pessary insertion, like the results of our study.^(47)^

The impact of cervical pessary on perinatal outcomes, including fetal and neonatal demise, has been widely discussed. Although our meta-analysis found no significant difference in outcomes between the use of cervical pessary and standard care. A study conducted by Liem et al.^(10)^ demonstrated that patients with a cervical length of 38mm or less who used a cervical pessary had significantly fewer instances of poor perinatal outcomes. Nevertheless, a meta-analysis specifically focusing on perinatal morbidity and mortality risks associated with cervical pessary placement suggested that there was no significant association.^(34)^

This study has several limitations. Firstly, there was moderate to high heterogeneity observed in certain outcomes analyzed, such preterm birth before 37 weeks of gestation. However, we conducted leave-one-out sensitivity analyses and obtained consistent results after excluding each study from the analysis. Secondly, the lack of patient-level data on the use of progesterone hindered a subgroup analysis of patients who received this therapy. Moreover, not all studies included in the analysis had consistent definitions of a short cervix as 25mm or less, or of preterm delivery as spontaneous. Thirdly, while this study represents the largest pooled analysis of patients treated with cervical pessary in singleton and twin pregnancy, it remains underpowered to examine long-term adverse neonatal endpoints and the use of pessary in women with suspected vaginal or cervical infections.

## Conclusion

In this meta-analysis, the use of cervical pessary did not show superiority when compared to progesterone in preventing preterm delivery. However, the use of cervical pessary was associated with decreased risk of preterm delivery before 37 weeks in patients with a short cervix in twin pregnancies, as compared to standard care, in this case without progesterone. Therefore, further studies comparing cervical pessary to vaginal progesterone may be necessary to provide a more comprehensive understanding of the potential benefits or disadvantages of cervical pessary.
